# Cancer of Pylorus, Etc.

**Published:** 1875-07

**Authors:** J. H. Etheridge, I. N. Danforth

**Affiliations:** Chicago; Chicago


ARTICLE V.

### CANCER OF THE PYLORUS — DEATH — AUTOPSY —MICROSCOPY AND PATHOLOGY.

BY J. H. ETHERIDGE, M.D., CHICAGO.

Mrs. —— ——, æt. 63, had complained many months of a gradually increasing dyspepsia, prior to New Years last. Twice or three times she had been prescribed for, for her supposed indigestion. Her malady first manifested itself by acid eructation and distress in the region of the stomach. These gradually increased till she had reduced the range of her dietary to a very few articles of food. The eructations became more copious, coming on at first an hour or two after meals. Soon she began to have them after retiring at night, and her rest was very much broken.

Jan. 16, 1875. I saw the patient, first time, to-day; prescribed fifteen drops dilute hydrochloric acid before meals, and pepsine and pancreatine after meals.

Jan. 18. No better. Is kept awake so much at night, by acid eructations, that her strength is decreasing; sleeps not more than two hours during night; when she does sleep she is suddenly awakened by strangling from the large amount of eructation.

Gave fifteen grs. subnitrate of bismuth three times a day, and at bedtime ten grs. of chloral and eight grs. of bromide of potassium. Immediately after this, her nights became better; the eructations varied in amount—some days being much less than before, on other days they were as copious as ever. The gastric distress varied greatly also. She ate more—bore more food. Some days she even got along without a symptom—and others were full enough of distress.

Jan. 29. 11 P. M. Found her troubled with vomiting, and it continued for a few hours. No apparent cause could be detected—no irregularity or indulgence in food could be found to account for it. Mustard to the epigastrium was followed by cessation of this troublesome symptom. Suspicion of malignancy was at once aroused, and subsequent consultation with Professor Byford, at the patient's request, decided me to call it cancer.

From this date the history of the case was the ordinary account of gastric malignant disease. Progressive emaciation, debility, and the complexion of the cancerous diathesis, were conspicuous. Vomiting, while giving carbolic acid (1 gr. four or six times a day), seemed to be controlled, and for nine or ten days she did not vomit at all.

Feb. 14. She recommenced vomiting, however, in spite of this drug, and was incessantly nauseated for many days. Opium to partial narcosis was thereupon exhibited, with the effect of holding in check this frightful symptom.

For five weeks and two days she was fed by rectal injections four or six times daily, of beef essence, wine and milk.

Throughout, she had *none* of the pain in the gastric region which is so generally present as to be termed *characteristic pain.*

Eleven days before death she was in the ataxic, crocidismic, carphologic condition seen in the third week of dothmenteritis. Regular omichesis, one of the *occasional* accompaniments of ischuria vesicalis, was a characteristic of the condition of the latter days of the patient, although she was, during that time, suffering from a continually distended bladder.

The condition of the bowels presented naught worthy of notice till six days before her demise. At that time there were five or six fæcal dejections daily for three consecutive days. During this time she must have voided more than a *chamber vessel full of solid feculent matter*. Prior to this, the bowels moved very irregularly —sometimes daily and sometimes twice a week, movements being small in amount, and ,generally were the remnants of the imperfectly digested clysters.

The skin, tongue, thoracic and pelvic organs presented nothing worthy of mention.

I have reserved one feature of this case, antedating, many years, the condition now under consideration, of purpose to call special attention to it as a probable precursor of malignancy. About thirty years ago, the patient was poisoned with arsenic, in partaking of some cold chicken that had been cooled, after cooking, in a new refrigerator, arsenic being absorbed by the fowl, in sufficient quantity to poison herself and family. Acute arsenical poisoning followed, the intensity being insufficient to produce death in any member of the family. Ever after that the patient suffered from gastric disorder, which had never before existed. During all these thirty years she could not eat certain articles of food without suffering ; at times she had epigastric pain and tenderness ; occasional vomiting also troubled her ; in short, all through these thirty years she was not as she used to be, prior to partaking of that cold chicken, because of chronic gastritis.

---

### PATHOLOGY AND MICROSCOPY.

By I. N. DANFORTH, M.D., Chicago

The pyloric orifice of the stomach was nearly closed by a dense, hard, constricting band. It was only by the exercise of considerable effort that I could force the tip of my little finger through it ; in fact, communication between the stomach and duodenum was practically cut off. Nearly the entire surface of the stomach was intensely inflamed—and, from the distended and tortuous state of the vessels, it was evident that this inflammation was no new thing.

Microscopic examination of sections of the proper walls of the stomach revealed nothing more than the ordinary inflammatory changes, namely, thickening or "hyperplasia" of the submucous connective tissue, and of the inter-follicular connective tissue ; and also the usual distension of the peptic glands by the rapid proliferation of their epithelial lining.

The indurated band surrounding the pylorus cannot be disposed of so easily. What is this indurated mass? Is it malignant in its character? or is it merely inflammatory new formation? or, lastly, was it originally inflammatory new formation, which at last suffered an invasion of cancer cells? Of course these questions cannot be answered absolutely, but I think we have a fair basis for the formation of an opinion.

The gross appearances of the mass are altogether indicative of cancer. It is dense, hard, unyielding, with that peculiarly "springy" or "hard-rubber" feel which we have all so many times noticed. Under the scalpel, it gives the regular orthodox sensation, as though one were cutting a piece of ill-formed cartilage—or rather a mass of dense fatty tissue, into which some stray cartilage had straggled. The cut surface shows the "pearly-blue" color, so characteristic of fresh young scirrhus. If the examination were not pushed beyond the naked-eye appearances, then, we should at once conclude that we were dealing with a case of unadulterated scirrhus. Immediately after the stomach was removed from the body, I incised the indurated mass, scraped from its cut surface a drop of "juice," and placed it under a small microscope. The characteristic "atypical cells" of Billroth were at once seen to be present, both by myself and several medical gentlemen in attendance, and we felt ourselves warranted in pronouncing the disease "*cancer.*" The further microscopic study of this case has been very interesting and instructive. I made forty or fifty thin sections in various directions, stained them with Thiersch's carmine, and then prepared them for mounting in damar. Of these specimens, I have mounted, and carefully studied nine; but as they are uniformly of substantially the same structure, perhaps I am now warranted in making a few observations upon them.

In the first place, the basis of every section examined seems to be fibres or fibre-cells; that is, spindle cells. The fibres are the small symmetrical fibres of inflammatory new formation; they are woven together precisely as they generally are in inflammatory new growth, and not after the "honey-combed" manner of the fibrous stroma of scirrhus. The spindle cells are exactly such as we should expect to find in an unsuspected mass of inflammatory induration; they are the ordinary uni-nucleated cells of connective tissue, which are undergoing a hyperplastic process.

But insinuated among these proliferating connective tissue cells, are occasional groups of cells of a far different nature, and of radically different appearance. I find here and there, in irregularly linear groups or rows, asymmetrical or atypical cells. Sometimes these cells occur in groups or "nests," similar to those which are found in schirrus of the breast; but they are generally arranged in linear rows, or "cylinders."

Another fact worth mentioning, and, indeed, quite important in connection with the history of the case, is this: That most of these atypical cells are half developed or young cells; and, from the manner in which they are huddled together in irregular groups, which include immense numbers, we are compelled to conclude that they were growing very rapidly at the time of the patient's death.

This case was doubtless, in the first instance, one of simple inflammatory induration; as such it for a long time remained. Meantime the whole stomach became gradually involved in the inflammatory process by simple communication from cell to cell. At last the indurated mass around the pylorus was invaded or "infiltrated" by true cancer cells. I think the term "invaded" expresses the real fact most nearly. The inflammatory induration was, from the outstart, a tissue of low type, and poorly able to resist an outside attack. But while the submucous connective tissue was undergoing this indurating process, the contiguous mucous layer and its epithelium was gradually yielding to the demoralizing influence of a diseased process. *Now, if an epithelial cell be in any degree estranged or provoked from its own proper law of growth, there is always danger that it may never return to its own proper law of growth; that is, that it may become, in its future generations,* ATYPICAL *or* LAWLESS—*and that is the very essence of malignancy.*

Just such an event, I think, did occur in this place. The inflammatory process was the primary process, commencing many years before death; the cancerous invasion only occurred after years of constant "teasing," probably not till the physiological integrity of the mucous tissue had been practically destroyed. The history of the case points to the same conclusion, and we are also borne out in this belief by authorities of extended observation and large experience.

This case demonstrates, in a striking manner, the strict truthfulness and importance of Mr. Arnott's observation, that thin sections, covering some considerable extent of surface, must be studied, if we would avoid errors. If the "scrapings" or "juice" alone of this specimen had been examined, we should have jumped at the conclusion that the case was primarily one of cancer. But when thin sections are examined, they tell not only the truth, but the *whole* truth; not only the present condition, but the past history of the case. It is precisely such cases as this that merit and reward the greatest amount of study; and it is only when careful clinical and microscopical studies go hand in hand, that the science of pathology is in reality greatly benefited.

